# Myocardial testosterone glucuronide and disease phenotypes in hypertrophic cardiomyopathy

**DOI:** 10.1093/eschf/xvag024

**Published:** 2026-01-16

**Authors:** Takenori Ikoma, Keitaro Akita, Kazuto Ohno, Terumori Satoh, Ryota Sato, Ariful Islam, Md Monirul Islam, Ryo Omagari, Kazuyuki Komatsu, Kyoko Unno, Keisuke Iguchi, Kenichiro Suwa, Atsushi Sakamoto, Tomoaki Kahyo, Itaru Takamisawa, Morimasa Takayama, Tomohiro Iwakura, Shuichiro Takanashi, Hirotomo Saitsu, Mitsutoshi Setou, Yuichiro Maekawa

**Affiliations:** Division of Cardiology, Internal Medicine III, Hamamatsu University School of Medicine, 1-20-1 Handayama, Chuo-ku, Hamamatsu 431-3192, Japan; Division of Cardiology, Department of Medicine, Massachusetts General Hospital, Harvard Medical School, Boston, MA, USA; Division of Cardiology, Internal Medicine III, Hamamatsu University School of Medicine, 1-20-1 Handayama, Chuo-ku, Hamamatsu 431-3192, Japan; Division of Cardiology, Internal Medicine III, Hamamatsu University School of Medicine, 1-20-1 Handayama, Chuo-ku, Hamamatsu 431-3192, Japan; Division of Cardiology, Internal Medicine III, Hamamatsu University School of Medicine, 1-20-1 Handayama, Chuo-ku, Hamamatsu 431-3192, Japan; Division of Cardiology, Internal Medicine III, Hamamatsu University School of Medicine, 1-20-1 Handayama, Chuo-ku, Hamamatsu 431-3192, Japan; Department of Cellular and Molecular Anatomy, Hamamatsu University School of Medicine, Hamamatsu, Japan; Department of Biochemistry and Microbiology, North South University, Dhaka, Bangladesh; Department of Cellular and Molecular Anatomy, Hamamatsu University School of Medicine, Hamamatsu, Japan; Department of Cellular and Molecular Anatomy, Hamamatsu University School of Medicine, Hamamatsu, Japan; Department of Biochemistry, Hamamatsu University School of Medicine, Hamamatsu, Japan; Division of Cardiology, Internal Medicine III, Hamamatsu University School of Medicine, 1-20-1 Handayama, Chuo-ku, Hamamatsu 431-3192, Japan; Division of Cardiology, Internal Medicine III, Hamamatsu University School of Medicine, 1-20-1 Handayama, Chuo-ku, Hamamatsu 431-3192, Japan; Division of Cardiology, Internal Medicine III, Hamamatsu University School of Medicine, 1-20-1 Handayama, Chuo-ku, Hamamatsu 431-3192, Japan; Division of Cardiology, Internal Medicine III, Hamamatsu University School of Medicine, 1-20-1 Handayama, Chuo-ku, Hamamatsu 431-3192, Japan; Department of Cellular and Molecular Anatomy, Hamamatsu University School of Medicine, Hamamatsu, Japan; Quantum Imaging Laboratory, Division of Research and Development in Photonics Technology and International Mass Imaging and Spatial Omics Center, Institute of Photonics Medicine, Hamamatsu University School of Medicine, Hamamatsu, Japan; Hypertrophic Cardiomyopathy Center, Department of Cardiology, Sakakibara Heart Institute, Fuchu, Japan; Hypertrophic Cardiomyopathy Center, Department of Cardiology, Sakakibara Heart Institute, Fuchu, Japan; Department of Cardiovascular Surgery, Sakakibara Heart Institute, Fuchu, Japan; Department of Cardiovascular Surgery, Sakakibara Heart Institute, Fuchu, Japan; Department of Biochemistry, Hamamatsu University School of Medicine, Hamamatsu, Japan; Department of Cellular and Molecular Anatomy, Hamamatsu University School of Medicine, Hamamatsu, Japan; International Mass Imaging and Spatial Omics Center, Institute of Photonics Medicine, Hamamatsu University School of Medicine, Hamamatsu, Japan; Division of Cardiology, Internal Medicine III, Hamamatsu University School of Medicine, 1-20-1 Handayama, Chuo-ku, Hamamatsu 431-3192, Japan

**Keywords:** Hypertrophic cardiomyopathy, Septal reduction therapy, Desorption electrospray ionization imaging mass spectrometry, Testosterone glucuronide

## Abstract

**Introduction:**

The relationship between myocardial lipid metabolism and disease phenotypes, genetic variation, and prognostic factors in patients with hypertrophic cardiomyopathy (HCM) remains largely unexplored. This study aimed to clarify how metabolic remodelling relates to disease phenotypes in HCM. We used desorption electrospray ionization imaging mass spectrometry (DESI-IMS) to compare myocardial lipid signatures in patients who required septal reduction therapy (SRT) and those who did not.

**Methods:**

In this prospective study, patients with HCM undergoing either right-ventricular biopsy or surgical septal myectomy were enrolled. Frozen 10 µm sections were analysed using DESI-IMS, and mass spectra were compared with volcano plots after signal-intensity normalization. Candidate ions showing |log_2_ fold change| ≥ 1 and *P* < .05 were annotated using the Human Metabolome Database.

**Results:**

Overall, 61 patients were analysed (34 [55.7%] were female; mean age 64.5 ± 15.9 years). Of these, 43 (70.5%) had obstructive HCM and 20 underwent SRT (SRT, *n* = 20; non-SRT, *n* = 41). The SRT group displayed a 3.3-fold higher signal for *m*/*z* 463.23, identified as testosterone glucuronide (*P* < .001). SRT remained the only predictor of elevated testosterone-glucuronide intensity in confirmatory regression (*β* = 1.991, *P* = .004), even after adjusting for age at diagnosis and the presence of hypertension, which significantly differed in baseline characteristics between the SRT and non-SRT groups.

**Conclusion:**

DESI-IMS revealed selective accumulation of testosterone glucuronide in hypertrophied myocardium from patients needing SRT, suggesting that androgen-linked metabolic remodelling plays a role in advanced obstructive physiology in HCM.

## Graphical Abstract

**Graphical Abstract:**
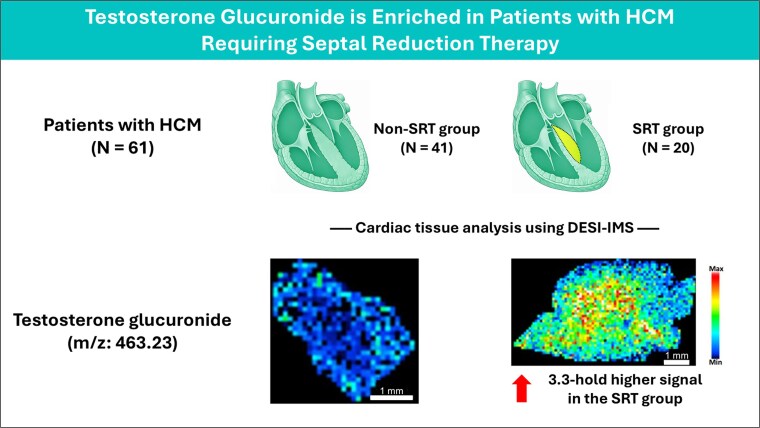
Among patients with HCM, desorption electrospray ionisation imaging mass spectrometry (DESI–IMS) of myocardial tissue (endomyocardial biopsy or myectomy samples) showed higher signal intensity of testosterone glucuronide (m/z 463.23) in those undergoing septal reduction therapy (SRT).

## Introduction

Hypertrophic cardiomyopathy (HCM) is one of the most common cardiomyopathies which presents diverse symptoms, morphological features, and cardiovascular events.^1^ HCM is also characterized by its genetic diversity, with approximately half of the patients who undergo genetic testing found to carry causal variants. However, this proportion varies by cohort, with reported penetrance rates of 46% and 18.4% in patients with a family history of HCM and the general population, respectively.^2–4^

HCM hearts exhibit unique metabolic and lipidomic features, characterized by a shift in primary energy substrate utilization from fatty-acid oxidation to glucose. This shift may impair the metabolic capacity of mitochondrial oxidation.^5,6^Recently, imaging mass spectrometry (IMS) has been employed to assess energy metabolism, helping to elucidate the cellular metabolic pathways in human tissue samples. We reported that the intensity of docosahexaenoic acid was significantly higher in endomyocardial biopsy (EMB) specimens from patients with HCM and severe disease conditions.^7^ In line with this finding, a recent experimental study indicates that a metabolic shift towards increased glucose utilization and aspartate-driven anabolic growth precipitates cardiac hypertrophy, whereas preservation of fatty-acid oxidation mitigates this pathological remodelling.^8^ However, links between myocardial metabolic remodelling and clinical symptoms in HCM remain poorly defined. We therefore aimed to delineate the relationship between myocardial metabolic profiles and disease phenotypes in HCM to advance diagnostic and therapeutic strategies.

## Methods

### Patients

We prospectively enrolled patients with HCM who underwent EMB or cardiac myectomy at Hamamatsu University Hospital and Sakakibara Heart Institute between July 2020 and February 2024. Patients were categorized into the following two groups: those who underwent septal reduction therapy [SRT; e.g. alcohol septal ablation (ASA) or surgical myectomy; SRT group] and those who did not (non-SRT group).

HCM was diagnosed based on echocardiographic evidence of left ventricular (LV) hypertrophy, defined as a maximum LV wall thickness of ≥15 mm in patients without and ≥13 mm in those with a family history of HCM. Patients with secondary cardiomyopathy exhibiting LV hypertrophy were excluded through medical history review, comprehensive physical examinations, and additional tests [e.g. cardiac magnetic resonance imaging (MRI), ^99m^technetium-pyrophosphate scintigraphy, and histopathological assessment of a tissue biopsy] as needed. Those with hypertension were also excluded when hypertensive LV hypertrophy was suspected, defined as a documented history of uncontrolled or long-standing hypertension with echocardiographic evidence of concentric LV hypertrophy proportionate to systemic haemodynamic load.^9^ The indication for SRT was determined by an intraventricular pressure gradient of ≥50 mm Hg under adequate medical therapy, along with the physician’s judgement based on the patient’s clinical condition. The choice between ASA and surgical myectomy was made at the discretion of the treating physicians.

Information on baseline characteristics was collected at the time of enrolment. Laboratory data, electrocardiogram (resting and ambulatory monitoring), echocardiography data, and cardiac MRI images were obtained as close to the enrolment date as possible. Genetic testing was conducted for eligible patients.

### EMB and cardiac myectomy

EMB samples were obtained from the right-ventricular septum using a bioptome. For patients undergoing ASA, myocardial specimens were obtained prior to the procedure. For those undergoing LV myectomy, the resected LV samples were acquired, and the precise excision site was determined intraoperatively at the discretion of the operating surgeon.

### Desorption electrospray ionization IMS

Biopsy and myectomy samples were promptly frozen in dry ice, then embedded in Super Cryoembedding Medium (Section-Lab, Japan), and stored at −80°C until sectioning. All samples were sectioned at a thickness of 10 μm with a cryostat (CM1950, Leica Biosystems, Wetzlar, Germany) at −20°C and placed on glass slides (Matsunami, Japan). Before the IMS acquisition, specimens were kept at room temperature for about 1 min to remove excess water. All experiments were conducted using a desorption electrospray ionization (DESI) source attached to a quadrupole time-of-flight mass spectrometer (Xevo G2-XS Q-TOF, Waters, Milford, MA, USA) in negative mode. The DESI-IMS mass spectra were externally calibrated with a 500 µM sodium formate solution in 90% 2-propanol. To maximize signal intensity from biopsy samples, DESI parameters were optimized as previously described with minor adjustments,^10^ including preliminary tuning using black ink (*m*/*z* 666.06) to enhance sensitivity for analyte imaging. A 98% methanol solution was used as the spray solvent at a flow rate of 3 μl/min. The following DESI and mass spectrometry parameters were used to acquire data in negative ionization mode: capillary voltage, 3.5 kV; cone voltage, 30 V; source temperature, 130°C; spatial resolution, 100 μm × 100 μm (X × Y); *m*/*z* range, 100–1000; scan rate, 200 μm/s; mass resolution, 20 000; mass window, 0.02 Da; and N_2_ gas pressure, 0.5 MPa.

For detailed analysis of DESI-IMS data, raw data were converted into imzML at first by HDImaging, and later to IMDX by IMDX converter (Shimadzu, Japan). Finally, IMDX data were analysed by IMAGEREVEAL (Shimadzu, Japan). Differences in the intensities between the two groups were analysed using a volcano plot (Microsoft Excel version 2019, Microsoft Corp, Redmond, WA, USA). The *x* axis represents the log_2_ of fold change in mass intensities of the same *m*/*z* between the groups, while the *y* axis shows −log_10_ of the *P* value derived from a *t*-test comparing the groups. Signals with *P* < .05 and a fold change of ≥2 between the SRT and non-SRT groups were selected as candidates for further identification. From these candidates, we confirmed that the signals originated from the sample area rather than background noise.

All candidate molecules corresponding to each targeted *m*/*z* were identified using the Human Metabolome Database (HMDB; https://www.hmdb.ca/) based on mass accuracy and known biological distributions. We corrected variations in signal intensity across different measurement sessions by normalizing the measurements using the average signal intensity of all signals in each session before analysis.

In addition, myocardial tissue was stained with haematoxylin and eosin (H&E), and the endocardial side was identified by an expert to examine whether the candidate metabolites were localized within the myocardial specimens.

### Genetic testing

Deoxyribonucleic acid was extracted from the patients’ blood samples. Based on previous reports, we conducted targeted sequencing using a custom panel for genes known to be causative of HCM.^3,11^ Patients with pathogenic or likely pathogenic variants in ClinVar database (https://www.ncbi.nlm.nih.gov/clinvar/) in genes encoding sarcomeric proteins were considered to have causative variants.

### Statistical analysis

All statistical analyses were performed using IBM SPSS Statistics for Windows, version 28.0.0.0 (IBM Corp., Armonk, NY, USA). Continuous variables are presented as the mean ± standard deviation and were compared using the unpaired *t*-test. Categorical variables are expressed as raw numbers and percentages and were analysed using Fisher’s exact test or χ^2^ test. For comparisons among three groups, the χ^2^ test was performed, and *post hoc* pairwise comparisons with Bonferroni correction were conducted when the overall test was significant. To investigate the association of candidate metabolites identified by DESI-IMS with factors other than SRT, we performed a multivariable analysis including SRT status and patient background variables that showed significant differences between the SRT and non-SRT groups. The correlation was evaluated using the Pearson correlation coefficient. Age-adjusted analyses were conducted using analysis of covariance for continuous variables, binary logistic regression for dichotomous variables, and ordinal logistic regression for New York Heart Association functional class. Statistical significance was set at *P* < .05.

### Consent to participate

The participants provided written consent before the study procedures began. This study was conducted in accordance with the regulations of the Declaration of Helsinki.

## Results

### Patient characteristics

Overall, 63 patients were enrolled in this study. Two patients were excluded because the obtained specimens were too small for IMS imaging, leaving 61 patients for the final analysis. Baseline characteristics are presented in Table 1. The cohort included 34 (55.7%) females, with a mean age of 64.5 ± 15.9 years. Forty-three (70.5%) patients had obstructive HCM (HOCM). Of the 51 patients who underwent the genetic testing, pathogenic variants were observed in 4 (7.8%) patients, including variants in *MYBPC3* (*n* = 2, 3.9%), *TNNI3* (*n* = 1, 2.0%), and *TNNT2* (*n* = 1, 2.0%).

**Table 1:** Baseline characteristics

	Non-SRT *n* = 41	SRT *n* = 20	*P*-value
HOCM	23 (56.1)	20 (100)	<.001
Age, years	67 ± 12	59 ± 21	.140
Age at diagnosis, years	65 ± 13	55 ± 24	.104
BMI, kg/m^2^	25.1 ± 3.8	25.3 ± 3.9	.843
Female	21 (51.2)	13 (65.0)	.309
NYHA class			.227
I	9 (22.0)	1 (5.0)	
II	22 (53.7)	14 (70.0)	
III	10 (24.4)	5 (25.0)	
IV	0 (0.0)	0 (0.0)	
Current smoker	9 (22.0)	3 (15.0)	.734
Family history of HCM	3 (7.3)	6 (30.0)	.048
Family history of SCD	2 (4.9)	3 (15.0)	.319
Medical history			
Atrial fibrillation	8 (19.5)	1 (5.0)	.249
Hypertension	30 (73.2)	9 (45.0)	.031
Diabetes	8 (19.5)	1 (5.0)	.249
Dyslipidaemia	24 (58.5)	11 (55.0)	.793
COPD	2 (4.9)	2 (10.0)	.591
Coronary artery disease	3 (7.3)	0 (0.0)	.544
Malignancy	4 (9.8)	1 (5.0)	1.000
Documentation of NSVT	3 (7.3)	0 (0.0)	.544
History of cardiopulmonary arrest	0 (0.0)	0 (0.0)	
History of unexplained syncope	5 (12.2)	3 (15.0)	1.000
Medication			
Na-channel blockers	3 (7.3)	11 (55.0)	<.001
Beta-blockers	25 (61.0)	18 (90.0)	.020
Calcium channel blockers	25 (61.0)	7 (35.0)	.057
ACE inhibitors or ARBs	16 (39.0)	3 (15.0)	.057
ARNI	2 (4.9)	0 (0.0)	1.000
Anticoagulation	8 (19.5)	1 (5.0)	.249
Amiodarone	1 (2.4)	1 (5.0)	1.000
SGLT2i	2 (4.9)	0 (0.0)	1.000
Operations and interventions			
Alcohol septal ablation	0 (0.0)	10 (50.0)	<.001
Surgical myectomy	0 (0.0)	11 (55.0)	<.001
Vital			
Systolic blood pressure, mm Hg	137 ± 26	127 ± 26	.134
Diastolic blood pressure, mm Hg	77 ± 16	68 ± 17	.052
Heart rate, b.p.m.	71 ± 14	69 ± 16	.553
Laboratory data			
NT-proBNP, pg/ml	1049 ± 1359	1301 ± 1010	.475
Troponin T, ng/ml	0.019 ± 0.012	0.017 ± 0.008	.511
CRP, mg/dl	0.14 ± 0.14	0.20 ± 0.53	.521
Haemoglobin, g/dl	14.0 ± 1.7	13.2 ± 1.5	.099
eGFR, ml/min/1.73 m^2^	58.9 ± 16.7	78.0 ± 41.7	.062
HbA1c, %	6.0 ± 0.7	5.7 ± 0.4	.079
LDL-C, mg/dl	100 ± 29	108 ± 30	.368
Echocardiographic variables			
Maximum LV wall thickness, mm	17.2 ± 2.5	18.4 ± 3.4	.133
LVEF, %	71 ± 6	68 ± 5	.059
LVDd, mm	42.9 ± 6.2	40.0 ± 5.2	.074
LVDs, mm	25.8 ± 4.4	24.8 ± 4.3	.407
LAD, mm	40.5 ± 6.5	40.7 ± 7.7	.902
LAVI, ml/m^2^	49.4 ± 21.5	54.1 ± 17.6	.408
*E*/*A*	0.97 ± 0.93	0.99 ± 0.55	.946
*E*/*e*′ (septal)	4.9 ± 1.7	4.8 ± 3.3	.897
*E*/*e*′ (lateral)	6.8 ± 1.9	5.7 ± 1.6	.040
LVOT-PG, mm Hg	31.3 ± 46.0	73.5 ± 31.6	<.001
MRI variables			
LV mass, g	125.7 ± 44.3	139.6 ± 51.7	.322
Apical aneurysm	4 (11.4)	2 (11.1)	1.000
LGE	18 (51.4)	11 (68.8)	.246
Pathogenic variants	2 (5.6)	2 (13.3)	.571
*MYBPC3*	1 (2.8)	1 (6.7)	.506
*TNNI3*	0 (0.0)	1 (6.7)	.294
*TNNT2*	1 (2.8)	0 (0.0)	1.000

Data are expressed as mean ± standard deviation or number (%).

Missing data for NT-proBNP (*n* = 1), troponin T (*n* = 16), CRP (*n* = 11), HbA1c (*n* = 1), LDL-C (*n* = 4), maximum LV wall thickness (*n* = 7), LAVI (*n* = 1), *E*/*A* (*n* = 5), *E*/*e*′ (septal; *n* = 1), *E*/*e*′ (lateral; *n* = 3), LVOT-PG (*n* = 2), LV mass (*n* = 9), apical aneurysm (*n* = 8), LGE (*n* = 10), and DNA mutation (*n* = 10).

ACE, angiotensin-converting enzyme; ARB, angiotensin receptor blocker; ARNI, angiotensin receptor–neprilysin inhibitor; BMI, body mass index; COPD, chronic obstructive pulmonary disease; CRP, C-reactive protein; *E*/*A*, ratio of early (*E*) to late (*A*) mitral inflow; *E*/*e*′, ratio of early mitral inflow (*E*) to early diastolic mitral annular velocity (*e*′); eGFR, estimated glomerular filtration rate; HbA1c, haemoglobin A1c; HCM, hypertrophic cardiomyopathy; HOCM, obstructive hypertrophic cardiomyopathy; LAD, left atrial diameter; LAVI, left atrial volume index; LDL-C, low-density lipoprotein cholesterol; LGE, late gadolinium enhancement; LV, left ventricle; LVDd, left ventricular end-diastolic dimension; LVDs, left ventricular end-systolic dimension; LVEF, left ventricular ejection fraction; LVOT-PG, left ventricular outflow tract pressure gradient; MRI, magnetic resonance imaging; NSVT, non-sustained ventricular tachycardia; NT-proBNP, N-terminal pro-B-type natriuretic peptide; NYHA, New York Heart Association; SCD, sudden cardiac death; SGLT2i, sodium-glucose cotransporter 2 inhibitor; SRT, septal reduction therapy.

Among all patients with HOCM, 20 underwent SRT (ASA *n* = 9, surgical myectomy *n* = 10, and both *n* = 1), while the others had no indication for the procedure. Accordingly, 20 and 41 patients were included in the SRT and non-SRT groups, respectively. Patients in the SRT group had a higher prevalence of a family history of HCM (*P* = .048) and a lower prevalence of hypertension (*P* = .031). A higher proportion of patients in the SRT group were also prescribed Na-channel blockers (*P* < .001) and beta-blockers (*P* = .020). (*Table 1*; comparisons among patients with non-obstructive HCM, HOCM without SRT, and HOCM with SRT are presented in Supplementary Table S1.)

### Desorption electrospray ionization IMS

**Figure 1:**
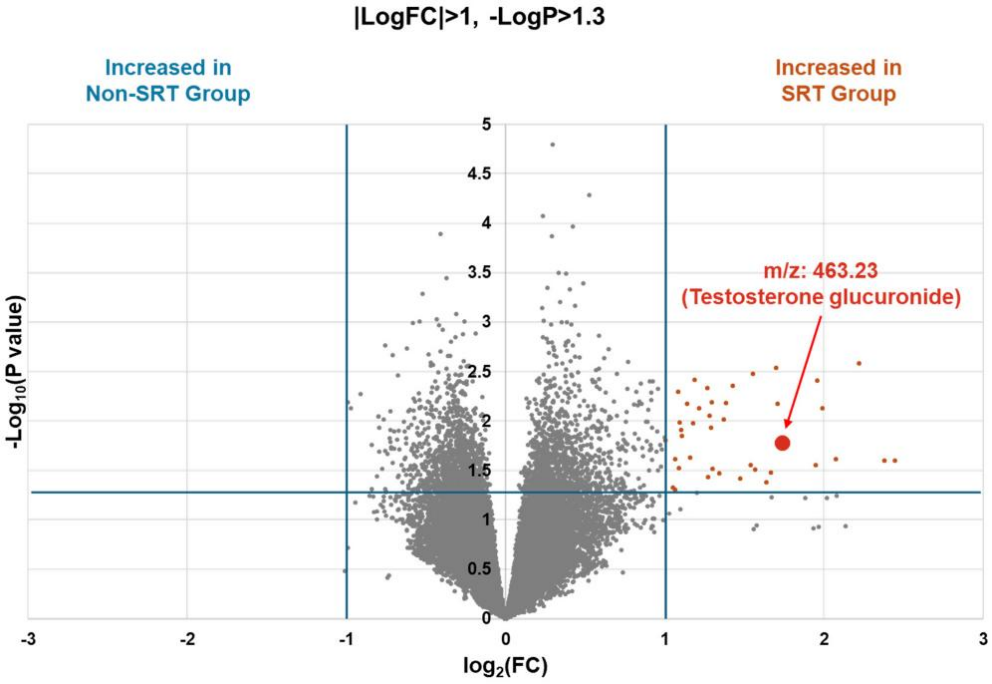
Volcano plot of the SRT and non-SRT groups. The volcano plot compares the SRT and non-SRT groups. SRT, septal reduction therapy

**Figure 2:**
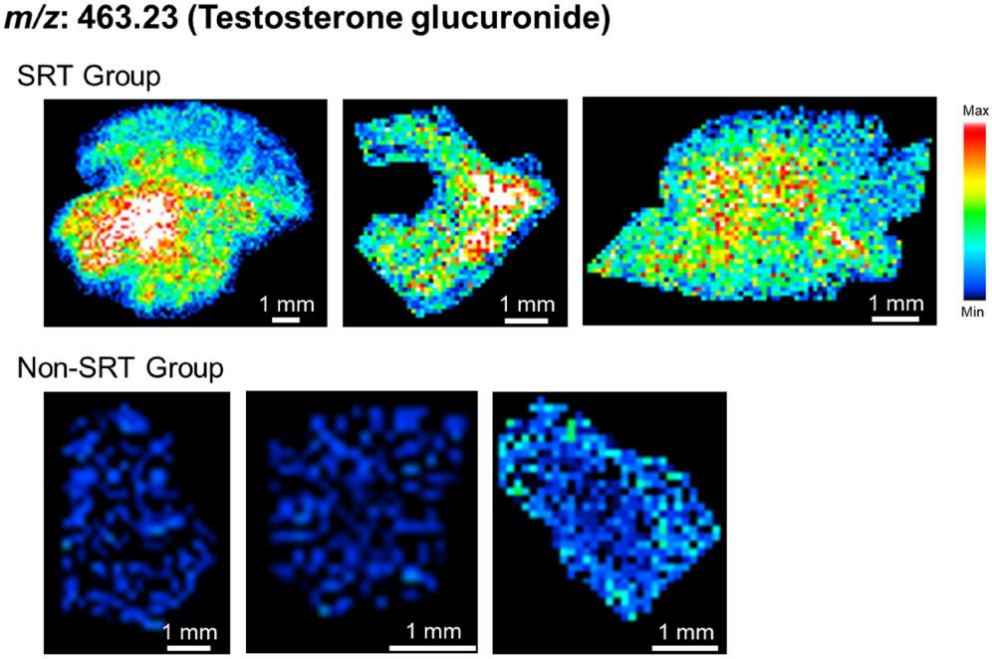
DESI-IMS imaging of testosterone glucuronide. Representative DESI-IMS images for *m/z* 463.23 (testosterone glucuronide). The top row shows three septal-myectomy samples obtained from the SRT group, while the bottom row displays three biopsy specimens from the non-SRT group. DESI-IMS, desorption electrospray ionization imaging mass spectrometry; SRT, septal reduction therapy

Using the volcano plot, we identified 40 signals with *P* < .05 and |log_2_ (fold change)| ≥ 1 via DESI-IMS (*Figure 1*). Each signal was then queried against the HMDB (see Methods), generating a list of putative metabolite matches. After excluding candidates unlikely to occur physiologically, testosterone glucuronide was the only compound with convincing evidence of *in vivo* presence (*Figure 2*). Multiple linear regression analysis was performed to identify factors associated with the increased signal intensity of testosterone glucuronide, including SRT implementation, age at diagnosis, and history of hypertension. Age was included because tissue testosterone concentrations have been reported to vary with age.^12^ The analysis revealed that SRT implementation was independently associated with increased signal intensity of *m*/*z* 463.23 (*P* = .004; *Table 2*).

**Table 2:** Multiple linear regression analysis of variables associated with predictors of the increase in high signal intensity of testosterone glucuronide

Variable	Beta	Standard error	*T* value	*P*-value
(Constant)	3.401	1.182	2.877	.006
Septal reduction therapies	1.991	0.672	2.965	.004
Age at diagnosis	−0.033	0.019	−1.791	.079
Hypertension	−0.292	0.695	−0.420	.676
*R* ^2^ = 0.248 (*P* < .001)				

**Figure 3:**
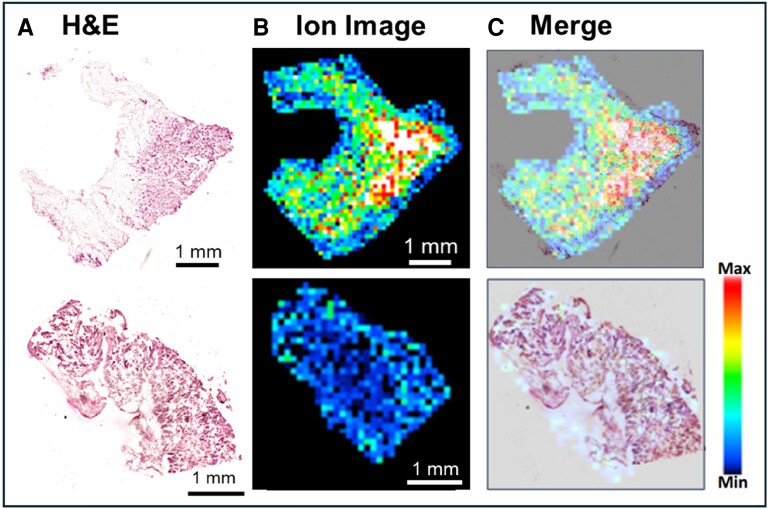
Spatial metabolomic profiling of an HCM septal-myectomy specimen using DESI-IMS. (A) H&E-stained sections of the resected interventricular septum tissue. (B) DESI-IMS ion map for *m*/*z* 463.23, assigned to testosterone glucuronide. (C) Overlay of the DESI-IMS signal-intensity map for testosterone glucuronide on the H&E section. DESI-IMS, desorption electrospray ionization imaging mass spectrometry; H&E, haematoxylin and eosin; SRT, septal reduction therapy

To assess whether there were any characteristic findings of testosterone glucuronide in the myocardial tissue obtained from septal myectomy, H&E staining was performed. The endocardium was identified in eight of the 10 patients who received septal myectomy. No substances exhibited a significant gradient extending from the endocardial side to other regions, including the fibrotic areas (*Figure 3*).

### Association between testosterone-glucuronide signal intensity and clinical factors in the SRT group

**Figure 4:**
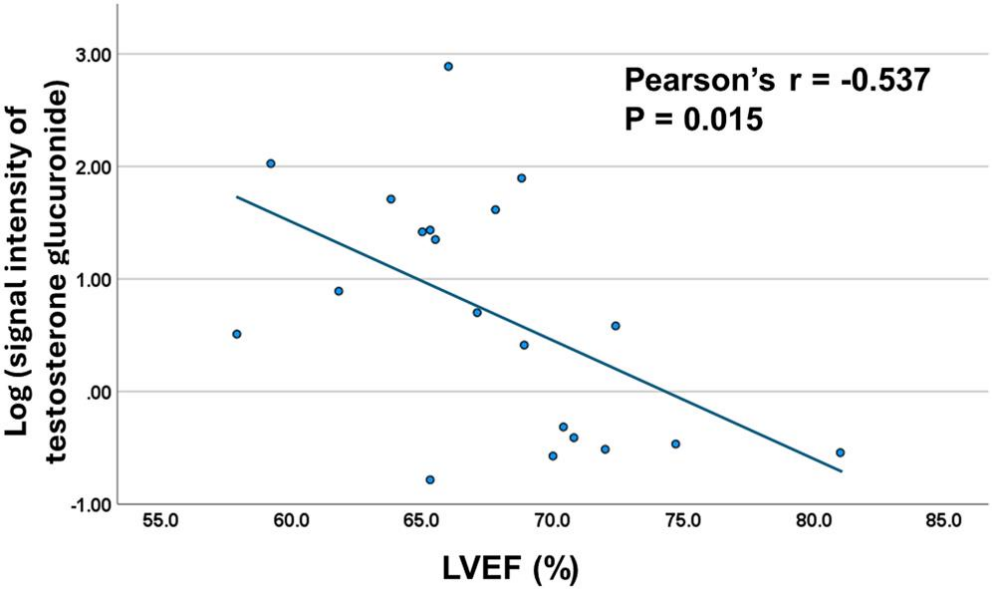
Association between the log-transformed signal intensity of testosterone glucuronide and LVEF in the SRT group. Scatter plot showing the association between LVEF and log-transformed DESI-IMS signal intensity of testosterone glucuronide. Each point represents an individual patient sample (*n* = 20). DESI-IMS, desorption electrospray ionization imaging mass spectrometry; LVEF, left ventricular ejection fraction; SRT, septal reduction therapy

We further investigated whether the signal intensity of testosterone glucuronide was associated with any clinical factors within the SRT group. Patients were categorized into two subgroups based on the median signal intensity of testosterone glucuronide. In unadjusted analyses, higher signal intensity was associated with younger age (*P* = .009), younger age at diagnosis (*P* = .018), a lower prevalence of dyslipidaemia (*P* = .005), lower heart rate (*P* = .023), lower haemoglobin A1c levels (HbA1c; *P* = .002), lower LV ejection fraction (LVEF; *P* = .021), and a higher prevalence of late gadolinium enhancement on MRI (*P* = .026; Supplementary Table S2). Because testosterone-glucuronide concentrations have been reported to vary with age in the liver,^12^ implying they may likewise differ within the heart, we carried out age-adjusted comparisons within the SRT cohort. After adjustment, higher signal intensity remained associated with a lower prevalence of dyslipidaemia (*P* = .042), lower heart rate (*P* = .009), lower HbA1c levels (*P* = .014), and lower LVEF (*P* = .049). There was no significant difference between female and male patients (*P* = .362). A scatter plot revealed a modest inverse relationship between the log-transformed signal intensity and LVEF (*r* = −0.537, *P* = .015; *Figure 4*).

## Discussion

This study revealed a markedly higher signal intensity of testosterone glucuronide in the SRT group, which is often considered to represent a more advanced obstructive physiology, although overall disease severity can vary and is not uniformly defined by SRT status.

Studies have indicated that sex hormones, particularly testosterone, play a vital role in maintaining cardiac health by mitigating both apoptotic cell death and excessive fibrotic remodelling.^13^ Testosterone preserves normal fatty-acid metabolism in cardiomyocytes through increased expression of peroxisome proliferator-activated receptor α (PPARα)^14^ and dampens oxidative stress by modulating nuclear factor kappa B.^15^ Consistent with these findings, pharmacological activation of PPARα using its agonist fenofibrate has been reported to attenuate cardiac hypertrophy and fibrosis.^16^ Additionally, the activation of the protein kinase B pathway enhances cell survival and growth signalling.^17^ Alongside these mechanistic insights, clinical studies have consistently reported improvements in skeletal muscle mass, protein synthesis, and baroreflex sensitivity under testosterone treatment.^18,19^ Moreover, recent findings on androgen receptor (AR) expression underscore the significance of androgen-mediated pathways in cardiac pathology. The myocardium derived from patients with aortic stenosis or HCM exhibits significantly higher AR levels than those in tissues obtained from patients with ischaemic cardiomyopathy, dilated cardiomyopathy, or non-failing hearts.^20^ Our findings extend these observations by demonstrating that testosterone-related metabolites accumulate in patients who undergo SRT and already display relatively low, although still ‘normal’, LVEF values. Because LVEF in HCM is frequently pseudo-normal or even supra-normal owing to hypercontractility and echocardiographic foreshortening, an elevated testosterone-glucuronide signal could mark the earliest phase of systolic deterioration or heightened metabolic stress within hypertrophied cardiomyocytes. Although the precise mechanisms remain unclear and require validation in animal models, two possibilities merit consideration. First, accumulation of testosterone-related metabolites may represent a compensatory response to functional impairment. Second, the effects may depend on the duration and magnitude of exposure to testosterone-related compounds. Prior studies have reported that anabolic-androgenic steroids can exacerbate hypertrophy, and upregulation of AR expression has been observed in HCM.^20,21^ Accordingly, while low or physiological doses of testosterone may exert cardioprotective effects, under conditions of elevated haemodynamic load (e.g. HCM meeting criteria for SRT) or early systolic decline, androgen actions may be amplified beyond the physiological range observed in healthy individuals, promoting further hypertrophy and functional deterioration. Further investigations into testosterone metabolism, including gene expression profiling and mutation screening of androgen pathway genes, are warranted.^22^

A notable strength of this study is the use of surgical myectomy specimens. The larger tissue volume allowed region-specific IMS analyses that are not feasible with EMB samples. Although no regionally restricted metabolites—including within fibrotic areas—were identified, cardiac MRI in symptomatic patients with HOCM undergoing septal reduction often reveals scarring in the basal septum, suggesting a predilection for fibrosis in this region.^23^ Studies involving larger patient cohorts and higher spatial resolution may help uncover metabolic gradients that parallel local variations in wall stress, perfusion, and fibrosis.

We detected no metabolites that correlated with known pathogenic sarcomere variants, a finding that may be attributed to limited sample size and the study’s focus on high-penetrance variants. Recent sequencing studies suggest that low-penetrance sarcomere-gene variants can exacerbate disease when combined with pathogenic alleles, underscoring the need for multi-omics approaches that integrate genome, transcriptome, proteome, and metabolome to clarify how genetic background influences metabolic reprogramming in HCM.^24^

This study had several limitations. First, our relatively small sample size limits the generalizability of findings and statistical power for subgroup analysis. Second, the classification of patients into SRT and non-SRT groups may have been influenced by the physician’s subjective judgement. Notably, the non-SRT group included 23 patients with HOCM who did not meet criteria for SRT. Thus, the SRT vs non-SRT comparison reflects procedural indication rather than a simple dichotomy of obstructive vs non-obstructive disease. Third, our results should be validated through additional research, including studies in animal models, to further confirm and expand upon our findings. Fourth, our cohort exhibited a relatively low prevalence of pathogenic variants. The population was characterized by an older median age at diagnosis (61.3 years), a low frequency of family history of HCM (14.8%), a high prevalence of concomitant hypertension (63.9%), and an absence of implantable cardioverter defibrillator implantation. Collectively, these features suggest that our cohort likely represents a population with a lower burden of pathogenic variants compared with previous reports.^25^

In conclusion, testosterone glucuronide is enriched in patients with HCM who require SRT. These findings support the involvement of androgen-related metabolic remodelling in advanced obstructive physiology and suggest that elevated levels of testosterone glucuronide may serve as an early indicator of LV systolic decline. Integrating spatial metabolomics with detailed genotyping in larger cohorts will be essential for validating these observations and developing targeted therapeutic strategies in HCM.

## Supplementary Material

xvag024_Supplementary_Data

## Declarations

### Data Availability

No data were generated or analysed for this manuscript. The data underlying this article cannot be shared publicly due to the privacy of individuals that participated in the study. The data will be shared on reasonable request to the corresponding author.

### Pre-registered Clinical Trial Number

None supplied.
