# Supplementary material for: Myocardial testosterone glucuronide and disease phenotypes in hypertrophic cardiomyopathy
Source: ESC Heart Fail. 2026 Jan 16;13(1):xvag024. doi: 10.1093/eschf/xvag024 (PMC13108264; doi:10.1093/eschf/xvag024)
Supplement: xvag024_Supplementary_Data [file xvag024_supplementary_data.docx]

**Supplemental Table 1. Comparison of baseline characteristics among HNCM, HOCM without SRT, and HOCM with SRT**

|  | HNCM  n = 18 | HOCM Non-SRT  n = 23 | HOCM SRT  n = 20 | P value |
| --- | --- | --- | --- | --- |
| Age, years | 66 ± 10 | 68 ± 13 | 59 ± 21 | 0.192 |
| Age at diagnosis, years | 62 ± 12 | 67 ± 13 | 55 ± 24 | 0.089 |
| BMI, kg/m^2^ | 25.5 ± 3.7 | 24.7 ± 3.8 | 25.3 ± 3.9 | 0.769 |
| Female | 6 (33.3) | 15 (65.2) | 13 (65.0) | 0.074 |
| NYHA Class |  |  |  | 0.266 |
| I | 3 (16.7) | 6 (26.1) | 1 (5.0) | – |
| II | 12 (66.7) | 10 (43.5) | 14 (70.0) | – |
| III | 3 (16.7) | 7 (30.4) | 5 (25.0) | – |
| IV | 0 (0.0) | 0 (0.0) | 0 (0.0) | – |
| Current smoker | 3 (16.7) | 6 (26.1) | 3 (15.0) | 0.613 |
| Family history of HCM | 2 (11.1) | 1 (4.3) | 6 (30.0) | 0.053 |
| Family history of SCD | 1 (5.6) | 1 (4.3) | 3 (15.0) | 0.397 |
| Medical history |  |  |  |  |
| Atrial fibrillation | 6 (33.3) | 2 (8.7) | 1 (5.0) | 0.028 |
| Hypertension | 11 (61.1) | 19 (82.6) | 9 (45.0)† | 0.036 |
| Diabetes | 5 (27.8) | 3 (13.0) | 1 (5.0) | 0.136 |
| Dyslipidaemia | 11 (61.1) | 13 (56.5) | 11 (55.0) | 0.925 |
| COPD | 0 (0.0) | 2 (8.7) | 2 (10.0) | 0.402 |
| Coronary artery disease | 1 (5.6) | 2 (8.7) | 0 (0.0) | 0.416 |
| Malignancy | 3 (16.7) | 1 (4.3) | 1 (5.0) | 0.295 |
| Documentation of NSVT | 2 (11.1) | 1 (4.3) | 0 (0.0) | 0.283 |
| History of cardiopulmonary arrest | 0 (0.0) | 0 (0.0) | 0 (0.0) | – |
| History of unexplained syncope | 2 (11.1) | 3 (13.0) | 3 (15.0) | 0.939 |
| Medication |  |  |  |  |
| Na-channel blockers | 1 (5.6) | 2 (8.7) | 11 (55.0)*† | < 0.001 |
| Beta-blockers | 12 (66.7) | 13 (56.5) | 18 (90.0) | 0.051 |
| Calcium channel blockers | 9 (50.0) | 16 (69.6) | 7 (35.0) | 0.075 |
| ACE inhibitors or ARBs | 7 (38.9) | 9 (39.1) | 3 (15.0) | 0.164 |
| ARNI | 2 (11.1) | 0 (0.0) | 0 (0.0) | 0.085 |
| Anticoagulation | 6 (33.3) | 2 (8.7) | 1 (5.0) | 0.028 |
| Amiodarone | 1 (5.6) | 0 (0.0) | 1 (5.0) | 0.532 |
| SGLT2i | 2 (11.1) | 0 (0.0) | 0 (0.0) | 0.085 |
| Operations and interventions |  |  |  |  |
| Alcohol septal ablation | 0 (0.0) | 0 (0.0) | 10 (50.0)*† | < 0.001 |
| Surgical myectomy | 0 (0.0) | 0 (0.0) | 11 (55.0)*† | < 0.001 |
| Vital signs |  |  |  |  |
| Systolic blood pressure, mm Hg | 133 ± 30 | 140 ± 23 | 127 ± 26 | 0.223 |
| Diastolic blood pressure, mm Hg | 74 ± 15 | 79 ± 17 | 68 ± 17 | 0.101 |
| Heart rate, bpm | 70 ± 15 | 73 ± 13 | 69 ± 16 | 0.678 |
| Laboratory data |  |  |  |  |
| NT-proBNP, pg/mL | 1158 ± 1309 | 964 ± 1420 | 1301 ± 1010 | 0.690 |
| Troponin T, ng/mL | 0.025 ± 0.014 | 0.015 ± 0.008 | 0.017 ± 0.008 | 0.033 |
| CRP, mg/dL | 0.12 ± 0.13 | 0.14 ± 0.15 | 0.20 ± 0.53 | 0.805 |
| Haemoglobin, g/dL | 14.3 ± 1.5 | 13.7 ± 1.8 | 13.2 ± 1.5 | 0.120 |
| eGFR, mL/min/1.73 m^2^ | 58.8 ± 17.8 | 59.0 ± 16.2 | 78.0 ± 41.7 | 0.048 |
| HbA1c, % | 6.2 ± 0.9 | 5.9 ± 0.6 | 5.7 ± 0.4 | 0.103 |
| LDL-C, mg/dL | 105 ± 28 | 96 ± 30 | 108 ± 30 | 0.433 |
| Echocardiographic variables |  |  |  |  |
| Maximum LV wall thickness, mm | 17.3 ± 2.7 | 17.1 ± 2.5 | 18.4 ± 3.4 | 0.324 |
| LVEF, % | 67 ± 6 | 74 ± 5* | 68 ± 5† | < 0.001 |
| LVDd, mm | 46.2 ± 4.9 | 40.3 ± 5.9* | 40.0 ± 5.2* | < 0.001 |
| LVDs, mm | 28.8 ± 3.7 | 23.5 ± 3.4* | 24.8 ± 4.3* | < 0.001 |
| LAD, mm | 41.4 ± 6.7 | 39.8 ± 6.3 | 40.7 ± 7.7 | 0.755 |
| LAVI, mL/m^2^ | 49.5 ± 25.1 | 49.4 ± 18.7 | 54.1 ± 17.6 | 0.712 |
| E/A | 0.95 ± 0.37 | 0.99 ± 1.20 | 0.99 ± 0.55 | 0.986 |
| E/e’ (septal) | 5.3 ± 2.2 | 4.6 ± 1.2 | 4.8 ± 3.3 | 0.603 |
| E/e’ (lateral) | 7.0 ± 1.6 | 6.6 ± 2.2 | 5.7 ± 1.6 | 0.099 |
| LVOT-PG, mm Hg | 14.1 ± 28.3 | 43.2 ± 52.3 | 73.5 ± 31.6* | < 0.001 |
| MRI variables |  |  |  |  |
| LV mass, g | 126.8 ± 50.3 | 124.7 ± 39.4 | 139.6 ± 51.7 | 0.610 |
| Apical aneurysm | 4 (23.5) | 0 (0.0) | 2 (11.1) | 0.090 |
| LGE | 12 (70.6) | 6 (33.3) | 11 (68.8) | 0.043 |
| Pathogenic variants | 1 (6.3) | 1 (5.0) | 2 (13.3) | 0.636 |
| *MYBPC3* | 0 (0.0) | 1 (5.0) | 1 (6.7) | – |
| *TNNI3* | 0 (0.0) | 0 (0.0) | 1 (6.7) | – |
| *TNNT2* | 1 (6.3) | 0 (0.0) | 0 (0.0) | – |

Data are expressed as mean ± standard deviation or number (%). When the overall test was significant, post hoc pairwise comparisons with Bonferroni correction were performed. *P < 0.0167 vs HNCM group; †P < 0.0167 vs HOCM non-SRT group (after Bonferroni correction).

Missing data for NT-proBNP (n = 1), Troponin T (n = 16), CRP (n = 11), HbA1c (n = 1), LDL-C (n = 4), maximum LV wall thickness (n = 7), LAVI (n = 1), E/A (n = 5), E/e’ (septal) (n = 1), E/e’ (lateral) (n = 3), LVOT-PG (n = 2), LV mass (n = 9), apical aneurysm (n = 8), LGE (n = 10), and DNA mutation (n = 10).

Abbreviations: ACE, angiotensin-converting enzyme; ARB, angiotensin receptor blocker; ARNI, angiotensin receptor–neprilysin inhibitor; BMI, body mass index; COPD, chronic obstructive pulmonary disease; CRP, C-reactive protein; E/A, ratio of early (E) to late (A) mitral inflow; E/e’, ratio of early mitral inflow (E) to early diastolic mitral annular velocity (e’); eGFR, estimated glomerular filtration rate; HbA1c, haemoglobin A1c; HCM, hypertrophic cardiomyopathy; HNCM, hypertrophic non-obstructive cardiomyopathy; HOCM, hypertrophic obstructive cardiomyopathy; LAD, left atrial diameter; LAVI, left atrial volume index; LDL-C, low-density lipoprotein cholesterol; LGE, late gadolinium enhancement; LV, left ventricle; LVDd, left ventricular end-diastolic dimension; LVDs, left ventricular end-systolic dimension; LVEF, left ventricular ejection fraction; LVOT-PG, left ventricular outflow tract pressure gradient; MRI, magnetic resonance imaging; NSVT, non-sustained ventricular tachycardia; NT-proBNP, N-terminal pro-B-type natriuretic peptide; NYHA, New York Heart Association; SCD, sudden cardiac death; SGLT2i, sodium-glucose cotransporter 2 inhibitor; SRT, septal reduction therapy.

**Supplemental Table 2. Comparison of baseline characteristics between patients with high versus low testosterone glucuronide signal intensity in the SRT group (unadjusted for age)**

|  | **Low**  **n = 10** | **High**  **n = 10** | **P value** |
| --- | --- | --- | --- |
| Age, years | 72 ± 7 | 47 ± 24 | 0.009 |
| Age at diagnosis, years | 68 ± 9 | 42 ± 28 | 0.018 |
| BMI, kg/m^2^ | 25.4 ± 4.2 | 25.1 ± 3.8 | 0.894 |
| Female | 7 (70.0) | 6 (60.0) | 1.000 |
| NYHA Class |  |  | 0.139 |
| I | 1 (10.0) | 0 (0.0) |  |
| II | 5 (50.0) | 9 (90.0) |  |
| III | 4 (40.0) | 1 (10.0) |  |
| IV | 0 (0.0) | 0 (0.0) |  |
| Current smoker | 2 (20.0) | 1 (10.0) | 1.000 |
| Family history of HCM | 2 (20.0) | 4 (40.0) | 0.628 |
| Family history of SCD | 1 (10.0) | 2 (20.0) | 1.000 |
| Medical history |  |  |  |
| Hypertension | 4 (40.0) | 5 (50.0) | 1.000 |
| Diabetes | 1 (10.0) | 0 (0.0) | 1.000 |
| Dyslipidaemia | 9 (90.0) | 2 (20.0) | 0.005 |
| History of unexplained syncope | 2 (20.0) | 1 (10.0) | 1.000 |
| Vital |  |  |  |
| Systolic blood pressure, mm Hg | 137 ± 27 | 116 ± 20 | 0.058 |
| Diastolic blood pressure, mm Hg | 74 ± 20 | 62 ± 11 | 0.135 |
| Heart rate, bpm | 77 ± 17 | 61 ± 11 | 0.023 |
| Laboratory data |  |  |  |
| NT-proBNP, pg/mL | 1017 ± 723 | 1557 ± 1192 | 0.256 |
| Troponin T, ng/mL | 0.015 ± 0.008 | 0.020 ± 0.009 | 0.341 |
| CRP, mg/dL | 0.08 ± 0.06 | 0.29 ± 0.71 | 0.414 |
| Haemoglobin, g/dL | 13.5 ± 1.2 | 13.0 ± 1.8 | 0.447 |
| eGFR, mL/min/1.73 m^2^ | 63.2 ± 13.8 | 92.7 ± 54.8 | 0.130 |
| HbA1c, % | 6.0 ± 0.4 | 5.4 ± 0.2 | 0.002 |
| LDL-C, mg/dL | 102 ± 25 | 117 ± 36 | 0.350 |
| Echocardiographic variables |  |  |  |
| Maximum LV wall thickness, mm | 17.1 ± 2.7 | 19.8 ± 3.5 | 0.072 |
| LVEF, % | 70.3 ± 6.0 | 65.0 ± 2.9 | 0.021 |
| LVDd, mm | 39.9 ± 2.7 | 40.0 ± 7.1 | 0.971 |
| LVDs, mm | 24.2 ± 3.1 | 25.5 ± 5.3 | 0.515 |
| LAD, mm | 42.8 ± 8.8 | 38.6 ± 6.1 | 0.227 |
| LAVI, mL/m^2^ | 59.6 ± 17.7 | 49.3 ± 16.9 | 0.213 |
| E/A | 0.77 ± 0.30 | 1.21 ± 0.66 | 0.070 |
| E/e’ (septal) | 5.6 ± 4.7 | 4.1 ± 0.9 | 0.358 |
| E/e’ (lateral) | 5.3 ± 1.3 | 6.1 ± 1.8 | 0.303 |
| LVOT-PG, mm Hg | 85.2 ± 26.3 | 61.8 ± 33.4 | 0.098 |
| MRI variables |  |  |  |
| LV mass, g | 132.8 ± 57.0 | 145.6 ± 49.1 | 0.627 |
| Apical aneurysm | 1 (12.5) | 1 (10.0) | 1.000 |
| LGE | 3 (37.5) | 8 (100.0) | 0.026 |
| Pathogenic variants | 0 (0.0) | 2 (28.6) | 0.200 |

Data are expressed as mean ± standard deviation or number (%).

Missing data for NT-proBNP (n = 1), Troponin T (n = 7), CRP (n = 2), HbA1c (n = 1), LDL-C (n = 4), LAVI (n = 1), E/e’ (septal) (n = 1), E/e’ (lateral) (n = 1), LV mass (n = 3), apical aneurysm (n = 2), LGE (n = 4), pathogenic variants (n = 5).

Abbreviations: BMI, body mass index; CRP, C-reactive protein; E/A, ratio of early (E) to late (A) mitral inflow; E/e’, ratio of early mitral inflow (E) to early diastolic mitral annular velocity (e’); eGFR, estimated glomerular filtration rate; HbA1c, haemoglobin A1c; HCM, hypertrophic cardiomyopathy; LAD, left atrial diameter; LAVI, left atrial volume index; LDL-C, low-density lipoprotein cholesterol; LGE, late gadolinium enhancement; LV, left ventricle; LVDd, left ventricular end-diastolic dimension; LVDs, left ventricular end-systolic dimension; LVEF, left ventricular ejection fraction; LVOT-PG, left ventricular outflow tract pressure gradient; MRI, magnetic resonance imaging; NT-proBNP, N-terminal pro-B-type natriuretic peptide; NYHA, New York Heart Association; SCD, sudden cardiac death; SRT, septal reduction therapy.
